# Effect of Myrtenol and Its Synergistic Interactions with Antimicrobial Drugs in the Inhibition of Single and Mixed Biofilms of *Candida auris* and *Klebsiella pneumoniae*

**DOI:** 10.3390/microorganisms10091773

**Published:** 2022-09-02

**Authors:** Angela Maione, Alessandra La Pietra, Elisabetta de Alteriis, Aldo Mileo, Maria De Falco, Marco Guida, Emilia Galdiero

**Affiliations:** 1 Department of Biology, University of Naples ‘Federico II’, Via Cinthia, 80126 Naples, Italy; 2 National Institute of Biostructures and Biosystems (INBB), 00136 Rome, Italy; 3 Center for Studies on Bioinspired Agro-Environmental Technology (BAT Center), 80055 Portici, Italy

**Keywords:** mixed biofilm, *Candida auris*, *Klebsiella pneumoniae*, myrtenol, essential oil

## Abstract

The increased incidence of mixed infections requires that the scientific community develop novel antimicrobial molecules. Essential oils and their bioactive pure compounds have been found to exhibit a wide range of remarkable biological activities and are attracting more and more attention. Therefore, the aim of this study was to evaluate myrtenol (MYR), one of the constituents commonly found in some essential oils, for its potential to inhibit biofilms alone and in combination with antimicrobial drugs against *Candida auris*/*Klebsiella pneumoniae* single and mixed biofilms. The antimicrobial activity of MYR was evaluated by determining bactericidal/fungicidal concentrations (MIC), and biofilm formation at sub-MICs was analyzed in a 96-well microtiter plate by crystal violet, XTT reduction assay, and CFU counts. The synergistic interaction between MYR and antimicrobial drugs was evaluated by the checkerboard method. The study found that MYR exhibited antimicrobial activity at high concentrations while showing efficient antibiofilm activity against single and dual biofilms. To understand the underlying mechanism by which MYR promotes single/mixed-species biofilm inhibition, we observed a significant downregulation in the expression of *mrkA*, *FKS1*, *ERG11*, and *ALS5* genes, which are associated with bacterial motility, adhesion, and biofilm formation as well as increased ROS production, which can play an important role in the inhibition of biofilm formation. In addition, the checkerboard microdilution assay showed that MYR was strongly synergistic with both caspofungin (CAS) and meropenem (MEM) in inhibiting the growth of *Candida auris*/*Klebsiella pneumoniae*-mixed biofilms. Furthermore, the tested concentrations showed an absence of toxicity for both mammalian cells in the in vitro and in vivo *Galleria mellonella* models. Thus, MYR could be considered as a potential agent for the management of polymicrobial biofilms.

## 1. Introduction

The increase in antimicrobic resistance (AMR) for pathogenic microorganisms represents a serious problem for human health, constantly worsening with consequences such as higher medical costs, prolonged hospital stays, and increased mortality. The solution is not only the search for new antimicrobials but also the exploration of the synergistic interactions of already existing drugs with natural compounds such as plant products. Essential oils (EOs) show antibiotic, antifungal, insecticidal, and antiviral activities and are obtained from various plants through different techniques including fermentation, enfleurage, extraction, and steam distillation [1,2]. Their mechanism of action has been extensively reported in the literature showing that also minor components in EOs can play an important role in the antimicrobial, anti-inflammation, and anti-oxidant activity either alone or in combination with commercial agents, even if limited knowledge exists regarding their activity against biofilms and also host-cell cytotoxicity [3].

Myrtenol (MYR) is a bicyclic alcohol mono-terpene plant derivative with a pleasant aroma used in a wide range of cosmetic and non-cosmetic products. MYR is found in the essential oil of numerous medicinal plants such as *Myrtus communis* [4] and has been used for the treatment of anxiety, gastrointestinal pain, inflammations, and infections [5] with antimicrobial [6] and antioxidant [7] activities. Its antibiofilm potential has not yet been explored sufficiently. An ideal antibiofilm agent is not expected to affect the growth and metabolic activity of the organism in order to exclude the development of resistance. In recent years, reports on the anti-biofilm activity of plant extracts have been increasing. In our previous studies, we assessed the in vitro anti-biofilm potential activity of extracts of wild *Allium ursinum* and *Allium oschaninii* on mixed biofilm *C. albicans*/*K. pneumoniae* [8]; the inhibition of the mixed biofilm *Pseudomonas aeruginosa*/*Staphylococcus aureus* formation with limonene at sub-Minimum Inhibitory Concentration [9]; and the eradication capacity of *Lavandula angustifolia* essential oil, free or encapsulated in liposomes, on primary and persister-derived biofilms of *C. auris* [10], showing that several compounds of plant origin, such as essential oils, could represent a valid alternative to combat biofilm-derived infections.

The importance of *non-albicans Candida* such as *Candida auris*, a highly drug-resistant pathogen, has increased in recent years, and a dramatic increase in morbidity and mortality has been recorded, particularly affecting ICU (intensive care unit) patients, and exhibiting a high capacity for skin colonization, a characteristic that likely contributes to patient-to-patient transmission. Furthermore, this emerging pathogen can live on surfaces outside the human body, further complicating the management of these infections by healthcare facilities [11,12]. Great importance results from its diverse virulence factors and fitness attributes, such as resistance to the majority of antifungal drugs and to environmental stress, adherence, and biofilm formation, and finally, the production of the extracellular hydrolytic enzymes proteinase and phospholipase [13]. Even though *C. auris* does not undergo filamentation easily, and has a weaker biofilm-forming ability, it can persistently colonize dry or moist surfaces for more than 14 days with a complex three-dimensional biofilm structure [14,15].

The World Health Organization has created a list of priority pathogens for which new kinds of treatments are greatly needed, of which carbapenem-resistant *Enterobacteriaceae* is flagged as a critical priority [16]. *K. pneumoniae* is one of the well-known human nosocomial pathogens, which causes urinary tract infections, community-acquired pneumonia, and hepatic abscesses and has been a major cause of mortality among *Enterobacteriaceae*, with such cases related to drug resistance. *K. pneumoniae* tends to form biofilms, which permits the colonization of host tissues and indwelling medical devices, besides persisting in hostile environments, resisting antibiotics, and clearance by the host immune response [16,17].

Biofilms are communities of microorganisms attached to a surface living in a matrix of extracellular material derived both from the cells themselves and from the environment, and more than 65% of human microbial infections are related to biofilm formation on implanted biomaterials or host surfaces. *C. auris* and *K. pneumoniae* can colonize the same habitats. It is evident from several studies that microorganisms in biofilm mode are less susceptible to the traditionally used antimicrobial drugs compared to their planktonic counterparts. In particular, polymicrobial biofilms formed by fungal and bacterial species are difficult to treat and can ultimately influence disease severity by promoting intensified pathogenic phenotypes, including an increase in resistance to both host defenses and antimicrobial therapies Therefore, novel anti-biofilm compounds or new therapeutic strategies are urgently needed. The combination of existing drugs has become the main alternative method. So, in recent years, a synergistic strategy has often been used to solve difficult-to-treat infections. The target of novel therapy is to inhibit biofilm formation and virulence factor production instead of killing the microorganisms and excluding the selection pressure on them, thereby avoiding resistance development. However, little work has been reported on MYR as an antibiofilm compound.

So, this study aims to evaluate the effects of MYR against the mixed biofilm of *C. auris*/*K. pneumoniae* at sub-MICs in vitro and in vivo, particularly focusing on the combination of it with antifungal and antibacterial drugs, caspofungin and meropenem, respectively, so as to explore the synergistic interaction of this plant-derived compound with conventional antimicrobials against *C. auris* and *K. pneumoniae*.

## 2. Materials and Methods

### 2.1. Chemicals

1R-Myrtenol, 96% purity, was procured from Sigma-Aldrich (Sigma-Aldrich Co., St. Louis, MO, USA) and dissolved in 5% dimethyl sulfoxide (DMSO) and 2% Tween 80 at a final concentration of 100 mg mL^−1^. Stock solutions of antimicrobial drugs caspofungin (CAS) (Sigma-Aldrich Co., St. Louis, MO, USA), and meropenem (MEM) (TCI EUROPE N.V. Boerenveldseweg, Zwijndrecht, Belgium) were used in this study and were dissolved in 5% DMSO at 25 mg mL^−1^ and 20 mg mL^−1^, respectively.

### 2.2. Strains and Culture Conditions

References strains such as *Candida auris* DSM 21092 and the Gram-negative *Klebsiella pneumoniae* ATCC 13883 were used in this work. Both *C. auris* and *K. pneumoniae* were maintained in the laboratory on Tryptic Soy Agar and cultivated in Tryptic Soy Broth supplemented or not with 1% *w*/*v* glucose, respectively (VWR chemicals).

HaCAT cells (non-tumorigenic human keratinocyte cells) were obtained from the ATCC (American Type Culture Collection, Manassas, VA, USA). They were maintained in Dulbecco’s Modified Eagle Medium (DMEM, Sigma Aldrich), supplemented with 10% Fetal Bovine Serum, 1% L-glutamine, and 1% penicillin/streptomycin (Sigma Aldrich) in a humidified incubator at 37 °C and 5% CO_2_. Once 70–80% confluency was reached, the cells were detached with Trypsin/EDTA solution (Sigma Aldrich) and cultured into new flasks. The medium was replaced twice a week.

### 2.3. Determination of Minimum Inhibitory Concentration (MIC)

Antimicrobial susceptibility was evaluated by using the Clinical and Laboratory Standards Institute (CLSI M27-A3 and M07-A9) microdilution reference method [18,19] with few modifications [20]. Briefly, 100 µL of fungal or bacterial culture was diluted to a final concentration of 1 × 10^6^ cells mL^−1^ in TSB with or without glucose, respectively, and added into each well of a 96-well-microplate with MYR (range 25–200 μg mL^−1^), or CAS (range 0.1–1 μg mL^−1^) or MEM (range 0.1–1 μg mL^−1^). Microbial cultures were incubated at 37 °C for 24 h and microbial growth was determined at 590 nm wavelength with a microplate reader (SYNERGYH4 BioTek). MICs of drugs were determined as the lowest drug concentration that produced ≥90% inhibition of growth relative to growth control.

### 2.4. Time–Kill Curves

To determine the potency of the action in relation to the time of MYR against *C. auris* and *K. pneumoniae*, a time to kill was carried out, exposing the microorganisms to concentrations equivalent to MIC and 2xMIC during incubation in culture medium. After 0, 2, 4, 6, and 8 h, cells were collected [21], properly diluted, and plated on TSA plates. The mean CFU count was used to determine the viable cells. All the experiments were performed in triplicate in three independent experiments.

### 2.5. Biofilm Formation and Characterization

To form single or mixed biofilms, 96-well sterile flat-bottomed microplate was seeded with standard inoculum of test organisms (100 μL per well), followed by incubation for 24 h at 37 °C. Total biofilm mass was quantified using the crystal violet (CV) staining methodology, and absorbance was quantified at 570 nm using a microtiter plate reader as described previously [22,23]. Biofilms vital biomass was quantified by using the tetrazolium 2,3-bis (2-methoxy-4-nitro-5 sulfophenyl)-5-[(phenylamine) carbonyl]- 2H-hydroxide reduction assay (XTT) (Sigma-Aldrich, St. Louis, MO, USA) according to the manufacturer’s instructions. The absorbance of resulting solution was measured at 492 nm using microtiter plate reader [24].

The CFU assay was performed to better characterize mixed biofilm formation. Briefly, adhered biofilms were completely scraped and serially diluted in Phosphate Buffered Saline (PBS). Cell diluted suspension was spread on Rose Bengal Agar plate supplemented with chloramphenicol (for *C. auris*) and TSA agar plate supplemented with amphotericin B (for *K. pneumoniae*). The resulting CFU count of biofilm cells was calculated after 24 h incubation at 37 °C. Each assay was conducted two times in triplicate and mean log CFU was used to determine the viability [25].

### 2.6. Minimum Biofilm Inhibitory Concentration (MBIC)

The ability of MYR to prevent single or mixed biofilm formation was investigated as previously reported with minor modifications [26]. Mono- and polymicrobial biofilms were allowed to develop for 24 h at 37 °C as previously described, but in the presence of MYR, at concentrations ranging from 6.2 to 200 μg mL^−1^. After 24 h, residual biofilm biomass was quantified by CV staining, as reported above. The percentages of inhibition were calculated as: % biofilm reduction = Abs control − Abs sample/Abs control × 100 [27].

### 2.7. qRT-PCR Analysis

Cells of single or mixed biofilm grown together with myrtenol (12.5 μg mL^−1^) at 37 °C for 24 h were scraped and washed in PBS as previously reported [28]. Total RNA was isolated using Direct-zolTM RNA Miniprep Plus Kit (ZYMO RESEARCH) according to the manufacturer’s instructions, and cDNA was obtained by reverse transcriptase (Bio-Rad, Milan, Italy) reaction using 1 μg of RNA. qRT-PCR was performed with 1 × SensiFASTTM SYBR Green master mix (total volume of 10 μL) (Meridiana Bioline) in an AriaMx Real-Time PCR instrument (Agilent Technologies, Inc., Milan Italy) according to the manufacturer’s instructions. Fluorescence was measured using Agilent Aria 1.7 software (Agilent Technologies, Inc.). The expression of each gene was analyzed and normalized against the ACT1 gene and 16SrRNA using REST software (Relative Expression Software Tool, Weihenstephan, Germany, version 1.9.12) based on the Pfaffl method [29,30]. The primer sequences used are listed in Table 1.

### 2.8. Measurement of Intracellular ROS Levels and Mitochondrial Specific ROS Accumulation

Intracellular reactive oxygen species (iROS) were determined using the fluorescent dye 2′,7′-dichlorofluorescein diacetate (DCFH-DA) (Molecular Probes, Eugene, OR, USA), and mitochondrial-specific ROS were measured by MitoSOX Red (Molecular Probes). Briefly, dual-biofilm cells treated with and without 12.5 μg mL^−1^ of MYR after centrifugation at 13,000× *g* for 5 min, were treated with 10 mM H_2_DCFDA for 1 h, or 5 M MitoSOX Red (Molecular Probes), for 30 min at 37 °C. The fluorescent cells were measured with the FACS Verse microplate reader [15].

### 2.9. Cell Rescue Assay Using ROS Scavengers

For cell rescue assay, two antioxidants N-acetyl cysteine (NAC) and glutathione were used (Sigma-Aldrich, St. Louis, MO, USA). Briefly, 200 μM NAC or 32 μM glutathione was added to microbial cells for 30 min at 37 °C. After incubation with the scavenger, cells were allowed to form the biofilm in the presence of 12.5 μg mL^−1^ MYR at 37 °C, as previously described. After biofilm formation, the adherent cells were scraped and plated on TSA or Rose Bengal Agar plates plus chloramphenicol for *K. pneumoniae* and *C. auris*, respectively, and incubated overnight at 37 °C to determine the number of CFUs. The results were reported as percentages of survival using the following formula: ((CFU of the sample treated with the agent)/(CFU of non-treated control) × 100). The data represent the mean ± standard deviation for three independent experiments [31].

### 2.10. Checkerboard Microdilution Assay

The combined effect of MYR and antimicrobial drugs (CAS and MEM) on single and mixed biofilm cells were determined by checkerboard microtiter assay, as described previously [25]. Briefly, the FICI was calculated for each agent by dividing the inhibition concentration of the antifungal combination by its MIC value. The calculation formula of the FICI model is as follows: FICI = (Ac/Aa) + (Bc/Ba), where Ac and Bc are the MIC values of tested agents in combination, while Aa and Ba correspond to these values for single-agent A and B treatments. A FICI of ≤0.5 means synergy; 0.5 < FICI ≤ 4 means no interaction; FICI > 4 means antagonism. Experiments were performed in triplicate [32].

### 2.11. MTT Assay

MYR effect was assessed by 3-[4,5-dimethylthiazol-2-yl]-3,5 diphenyl tetrazolium bromide (MTT) assay, which allowed for the correlation of the concentration of formazan crystals with cell viability. HaCAT cells were seeded in a 96-well plate at a density of 3 × 10^3^ cell/well. After starvation, the cells were treated with several MYR concentrations ranging from 5 to 200 μg mL^−1^. Control cells were treated with vehicle (0.01% DMSO, 0.004% Tween 80). After 24 h of treatment, MTT solution was added to each well and the cells were incubated for 4 h in a humidified incubator at 37 °C and 5% CO_2_. Then, the medium was gently removed and replaced with DMSO to dissolve the formazan crystals. The absorbance of formazan crystal was measured at 570 nm with a microplate reader.

### 2.12. Galleria Mellonella Assays: Toxicity, Infection Rescue Assay

Toxicity assays using the *Galleria mellonella* were performed as previously described [33]. Twenty randomly chosen *G. mellonella* with bodyweights of approximately 300 mg were used for each test group. The experiments were performed in triplicate. MYR was inoculated into *G. mellonella* through the inferior left proleg at varying concentrations (25, 50, 100, and 200 μg mL^−1^). Larvae were monitored for survival over three days. Death was defined as a complete loss of mobility and lack of response to a physical stimulus using a plastic pipette tip.

The *G. mellonella* mixed infection model was performed as described previously [20]. The injected concentration was 1:1 of each pathogen to reach a final concentration of 1 × 10^6^ total cells into the larvae. For the killing assay, larvae were inoculated with an aliquot of 10 μL of 12.5 μg mL^−1^ myrtenol on the inferior left proleg on the opposite side of the pathogen injection site, either 2 h pre-infection (for prevention experiments) or 2 h post-infection (for treatment experiments). Five groups were controls: one group of untreated larvae, one group received PBS/PBS solution per leg, one group DMSO/Tween 80 and PBS, one group was injected with 10 μL of MYR in one leg and 10 μL PBS in the other, and one group of 1 × 10^6^ pathogens and PBS.

Larvae were incubated at 35 °C in plastic containers, and the number of dead larvae was scored daily over 3 days.

### 2.13. Statistical Analysis

GraphPad Prism Software (version 9.00 for Windows, GraphPad Software, La Jolla, CA, USA, www.graphpad.com, accessed on 15 July 2022) was used for data analysis. All experiments were performed in triplicate, and results were shown as mean values ± standard deviation (SD). Survival curves were plotted using the Kaplan–Meier method. One-way ANOVA with Dunnett post-test was used. For molecular analysis, *t*-test was used to evaluate the difference between treatments and control group. *p* < 0.05 was considered statistically significant.

## 3. Results

### 3.1. Susceptibility Testing of Planktonic Populations of C. auris and K. pneumoniae

As shown in Table S1, the MIC of MYR was 200 µg mL^−1^ for *K. pneumoniae,* showing the non-antibacterial nature of MYR against this Gram-negative strain, while the MIC of *C. auris* was instead 50 µg mL^−1^, showing a better antifungal activity of MYR.

**Figure 1:**
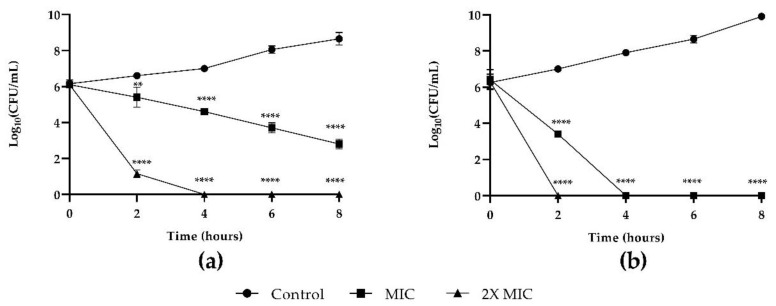
Time to kill of MYR against *C. auris* and *K. pneumoniae*: (**a**) growth curves generated using *C. auris* cells treated with 50 μg mL^−1^ (MIC) and 100 μg mL^−1^ (2× MIC) of MYR and (**b**) growth curves generated using *K. pneumoniae* cells treated with 200 μg mL^−1^ (MIC) and 400 μg mL^−1^ (2× MIC) of MYR ** = *p* < 0.01, **** = *p* < 0.0001 (Dunnett’s test).

MYR was tested on fungal and bacterial growth and results of time–kill studies are presented in Figure 1 (panels a and b, respectively). MYR proved the reduction in the fungal growth with the two used concentrations MIC and 2× MIC (panel a). At MIC concentration, the effect was not fungicidal within 8 h. At 2× MIC concentration, a CFU reduction (4 Log) was already observed after 2 h, and cells were killed after 4 h. Antibacterial activity of MYR at 2× MIC concentration had a rapid killing action after 2 h application when all cells were killed. At MIC concentration, *K. pneumoniae* started to decrease at 3 h, being completely killed at 4 h (panel b).

### 3.2. Effect of Myrtenol on C. auris and K. pneumoniae Biofilms

**Figure 2:**
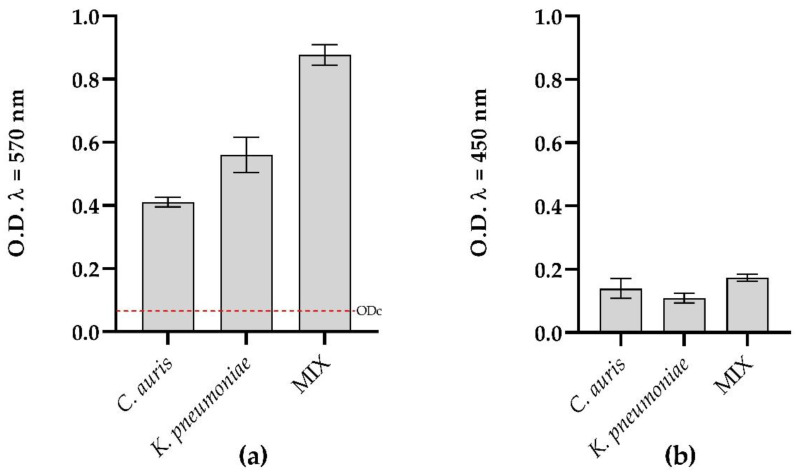
Characterization of single- and dual-species biofilm at 24 h with crystal violet (**a**) and XTT (**b**); Horizontal red line indicated ODc (ODcut = mean of negative control with addition of 3 times the SD).

Both microorganisms were able to form mono- and polymicrobial biofilms. Total biofilm biomass detected by Crystal Violet (Figure 2) demonstrated a strong ability to form biofilms over the surface of the microtiter plate after 24 h of incubation at 37 °C according to Stepanovic’s criteria [22]. Particularly, biomass increased in the dual-species biofilm of *C. auris*/*K. pneumoniae* and presented, as shown in Table 2, a predominance of *K. pneumoniae* over *C. auris* (94 vs. 6%, respectively). No significant differences in metabolic activity were found in both single and dual-species biofilms. (Figure 2, panel b).

For biofilm prevention, concentrations between 6.2 and 50 μg mL^−1^ of MYR were tested against *C. auris* and *K. pneumoniae* single and mixed biofilms.

**Figure 3:**
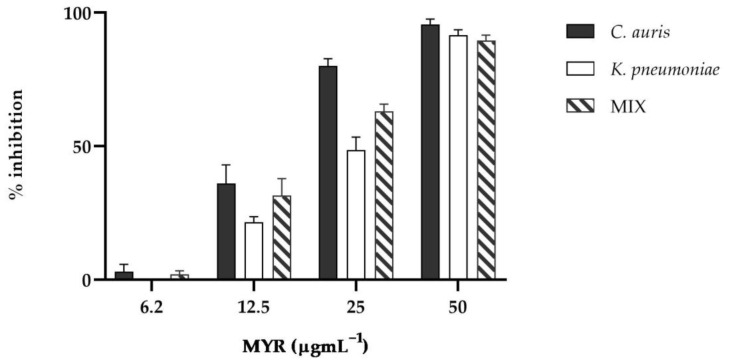
Antibiofilm activity of MYR on *C. auris* and *K. pneumoniae* quantified with crystal violet after 24 h.

As evidenced by crystal violet quantification, the single and mixed biofilms were significantly reduced in the presence of MYR as presented in Figure 3 (*p* < 0.0001). Already at a concentration of 12.5 µgmL^−^^1^, a reduction in biofilm mass was observed compared to the untreated control.

At the highest concentration tested, about 100% inhibition of biofilm formation was detected for both single and dual-species biofilm, confirming that strains were incapable of adhering to the wells and that myrtenol had the capacity to completely prevent biofilm formation.

### 3.3. Myrtenol Inhibited the Expression of Biofilm Formation Related Genes

In order to understand the molecular mechanism of inhibition of biofilm formation of MYR, the expression profiles of genes related to biofilm formation, control, and myrtenol-treated cells were studied using qRT-PCR analysis.

**Figure 4:**
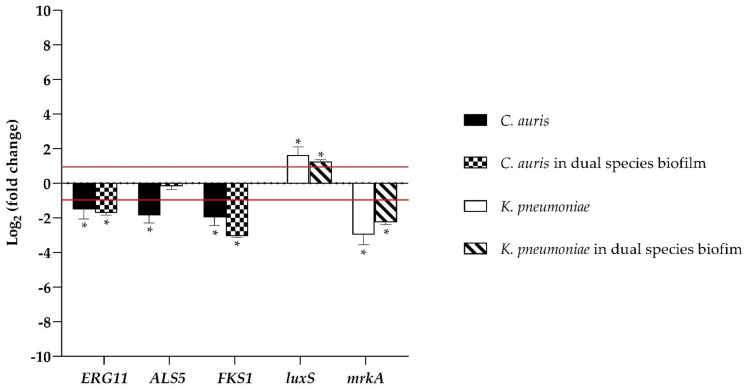
Real-time qPCR at 24 h of treatment with MYR at a concentration of 12.5 µgmL^−1^. Histograms show the mean ± SD of relative mRNA expression levels of virulence response genes. Horizontal red lines indicate fold change thresholds of 2 and 0.5, respectively. * = *p* < 0.05 (*t*-test).

Figure 4 represents the relative changes in the expression levels of selected genes of *C. auris* and *K. pneumoniae* indicated as fold expression values and normalized to each housekeeping gene *Actin* and *16S rRNA*, respectively, and calculated by the Rest method. The expressions of the *ERG11* gene, which is known to participate in ergosterol biosynthesis, and *FKS1,* associated with β-1,3-glucan synthase, a key enzyme to synthesize an essential component of the fungal cell wall, were remarkably downregulated during biofilm formation by myrtenol at a concentration of 12.5 µgmL^−1^ in both single and dual-species biofilms of *C. auris*. Because the adherence mechanism is an important virulence factor that is regulated by diverse genes and marks not only the beginning of infection by *Candida* spp. but also the persistence of the disease; the *ALS5* gene was detected here showing a downregulation in single treated biofilm, whereas it was not significantly affected by MYR in the mixed one.

The expression of the *mrkA* gene that encodes the major subunit of type 3 fimbriae and is essential in the initial stage of biofilm formation in *K. pneumonia* was significantly decreased by treatment in both single and dual-species biofilms, while *luxS*, one of the quorum sensing genes, was highly upregulated at 24 h.

### 3.4. Measuring ROS Production in Mixed Biofilm

**Figure 5:**
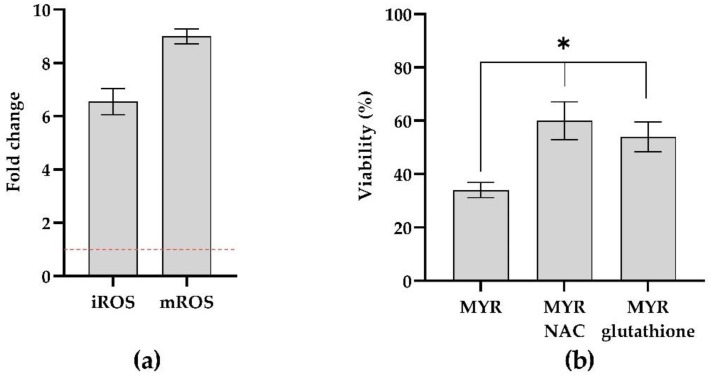
Induction of intracellular and mitochondrial ROS (**a**) and Cell Rescue Assay using ROS scavengers (**b**) on dual-species biofilm of *C. auris* and *K. pneumoniae* using MYR at the concentration of 12.5 μg mL^−1^. * = *p* < 0.05 (Dunnett’s test).

In order to establish the MYR effect at the subMIC concentration of 12.5 µg mL^−1^ on oxidative imbalance during mixed biofilm formation, the iROS and mROS were examined and iROS and mROS measurements in response to MYR exposure showed an increase of six and eightfold, respectively, when compared to untreated biofilms (Figure 5).

When the two blocking oxidative pathway compounds (NAC and glutathione) were used to evaluate the effect of oxidative stress generation on cell viability, we noticed that the pre-treatment with the two scavengers significantly increased the survival of the cells during biofilm formation (Figure 5, panels a,b).

### 3.5. Combined Antimicrobial Effect on Inhibition of Mixed Biofilms

**Figure 6:**
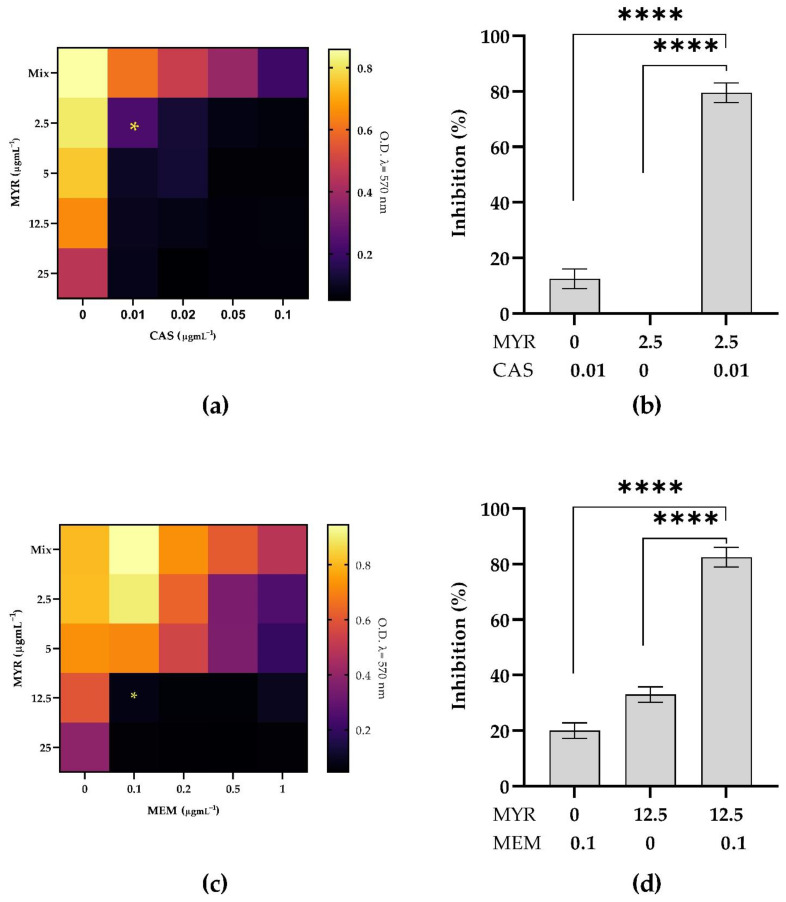
Combined activity of MYR with CAS (**a**,**b**) and MEM (**c**,**d**) against dual-species biofilm of *C. auris* and *K. pneumoniae*; panels (**a**,**c**) show checkerboard assay, and panels (**b**,**d**) show percentage of inhibition of biofilm formation. Stars indicated indicate synergy (FICI ≤ 0.5); **** = *p* < 0.0001 (Dunnett’s test).

The combined effect of MYR and CAS or MEM was studied on *C. auris* and *K. pneumoniae* mixed biofilms to detect possible synergistic effects (Figure 6). Figure 6 shows the checkboard assay results obtained with the combination of MYR with CAS or MEM and also reports the percentage of biofilm inhibition corresponding to the best combination of the two compounds giving a synergistic effect (FICI < −0.5). When MYR was tested in combination with CAS (Figure 6, panels a,b), the MIBC was obtained at 2.5 µg mL^−1^ MYR and 0.01 µg mL^−1^ CAS with a biofilm inhibition of 80%. In the case of MYR and MEM (Figure 6, panels c,d), the combination of 12.5 µg mL^−1^ MYR and 0.1 µg mL^−1^ MEM was the most effective.

### 3.6. Kaplan–Meier Survival Curves

**Figure 7:**
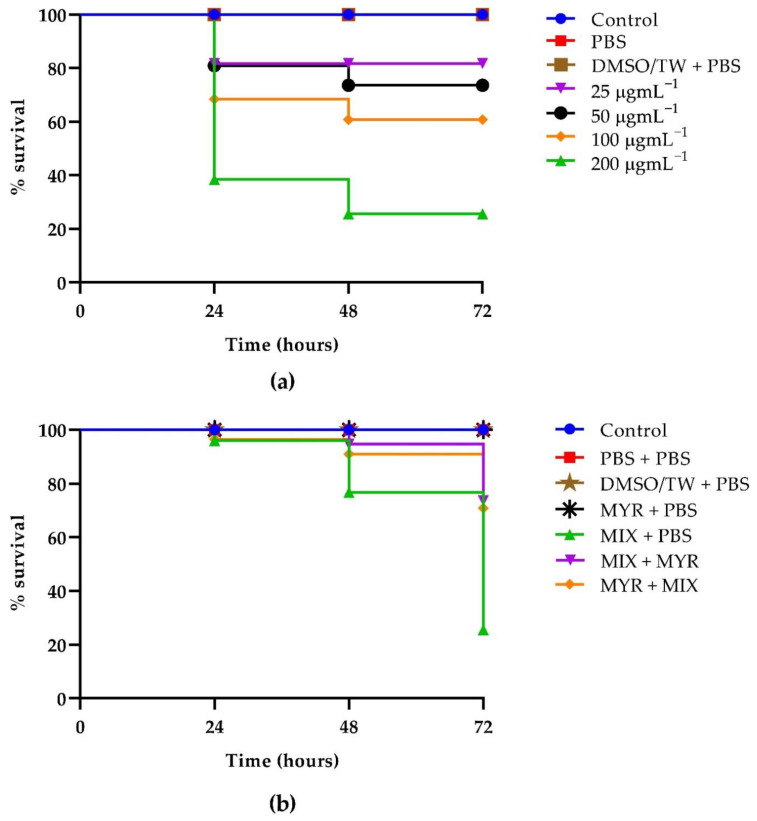
Kaplan–Meier plots of survival curves of *G. mellonella* larvae: (**a**) MYR Toxicity on *G. mellonella* larvae treated at the concentrations of 25, 50, 100, and 200 μg mL^−1^; (**b**) In vivo effectiveness of MYR (12.5 μg mL^−1^) on *G. mellonella* larvae infected with *C. auris* and *K. pneumoniae*.

First, we evaluated the toxicity of MYR in *G. mellonella*. The larvae were injected with varying MYR concentrations, and their survival was monitored for 3 days. MYR did not exert significantly toxic effects on the larvae when administered at concentrations of 25 µg mL^−1^ up to 72 h, since an 80% survival was observed (Figure 7, panel a). The toxicity increased in a dose-dependent manner showing only 30% survival after 24 h at the highest concentration tested. Finally, to further evaluate MYR as a potential anti-infective compound in vivo, we tested the concentration of 12.5 µg mL^−1^ on the mixed infection of *C. auris/K. pneumoniae* treating animals pre- and post-infection. Both treatments increased the viability of larvae by about 50% after 72 h compared to infected larvae untreated.

### 3.7. The Tested Concentrations of Myrtenol Exhibited No Considerable Cytotoxicity on In Vitro Human Keratinocyte Cells

**Figure 8:**
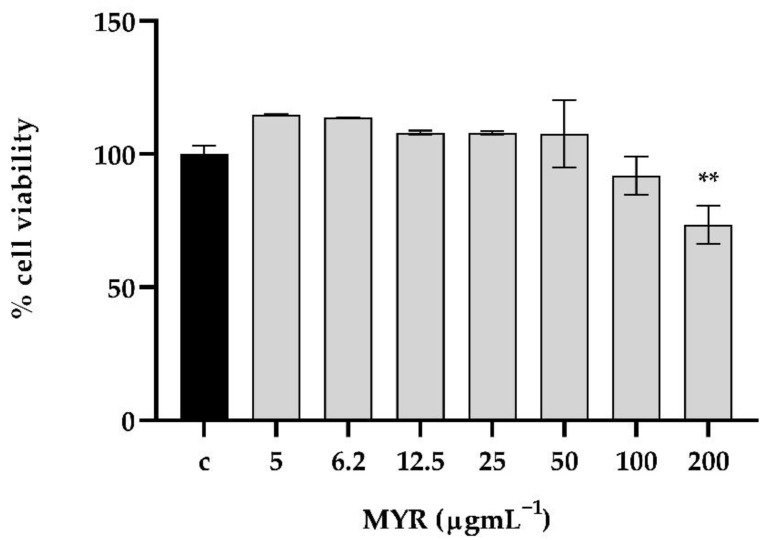
MTT assay after 24 h of exposure MYR (from 5 to 200 μg mL^−1^). MYR inhibited HaCAT cell proliferation at 200 μg mL^−1^ if compared to control (0.01% DMSO, 0.004% Tween 20). ** = *p* < 0.01 (Dunnett’s test).

MTT assay was performed to evaluate cell viability after 24 h of MYR exposure at increasing concentrations (from 5 to 200 μg mL^−1^). MYR did not affect cell viability at lower concentrations (from 5 to 100 μg mL^−1^). Only at 200 μg mL^−1^ did cell viability significantly decrease, compared to control group (Figure 8).

## 4. Discussion

The repurposing of drugs is an interesting strategy to discover new applications for drugs already in use, saving the high costs related to the discovery and development of new compounds. Therefore, this strategy has been widely applied against planktonic cells of several micro-organisms and scarcely reported on microbial biofilms. In addition, sometimes natural bioactive compounds fail to pass clinical trials due to high effective concentrations and/or toxic effects. To avoid this, recently, combinatorial drug therapy has brought greater advantages such as reduced toxicity, better efficacy with the reduction in antibiotic resistance, and, therefore, a greater potential compared to single drugs.

Both *C. auris* and *K. pneumoniae* are considered by European Centre for Disease Prevention and Control (ECDC) as emerging “superbugs” releasing clinical alerts [34]. *C. auris* can form biofilms that are difficult to damage, enhancing resistance to antifungal agents and host defenses, and weakening the effective treatment of this infection [35,36]. Biofilm formation is also a significant characteristic of *K. pneumoniae,* promoting their survival in hospital settings and increasing the probability of occasional nosocomial infections [37]. Therefore, screening for new antimicrobials and especially alternative modalities of therapy with new combinations of pre-existing drugs with improved modes of action without toxicity from various sources, including medicinal plants, is necessary. Medicinal plants are rich in diverse chemical structures, which warrants more thorough investigation as potential novel antimicrobial agents. Essential oils are complex mixtures of volatile compounds isolated by water distillation from a whole plant or individual parts and generally, their oxygenated compounds are responsible for the biological activity and generally attract more attention. MYR is a phytoconstituent present in *Myrtus communis* L. (*Myrtaceae*) essential oil with reported anxiolytic, anti-inflammatory, and gastroprotective [38,39] properties, but little knowledge of antibacterial and antibiofilm potential is described [40]. An ideal antibiofilm agent is not expected to affect the growth and metabolic activity of the organism in order to exclude the development of resistance. In a previous study, it was shown that myrtenol was effective in reducing the biofilm-forming ability of MRSA clinical isolates exhibiting a concentration-dependent antibiofilm without affecting growth [41].

Our study assessed, first of all, a strong biofilm formation for both the microorganisms under investigation, as similarly observed in our previous studies [20]. The activity of MYR against *C. auris* and *K. pneumoniae* planktonic cells was found only at high concentrations, but its effect on single and mixed biofilm prevention was significantly exerted at sub-MIC concentrations.

From a clinical point of view, the formation of mixed biofilms leads to negative consequences for health, being reservoirs of microorganisms and having extremely different properties from planktonic populations, especially a high resistance to numerous antimicrobial agents [28]. MYR displayed antibiofilm activities, as reported for EO components, possibly with a reduction in EPS production and with disturbance of membrane integrity. Our results, based on the gene expression analysis, demonstrated that the inhibition effect is also given by the downregulation of genes related to biofilm formation. *ERG11* and *FKS1,* involved in the primary mechanisms of action of antifungals (b-glucans biosynthesis and blocking and disturbance in ergosterol synthesis, respectively), and *ALS5,* involved in the initial stages of biofilm formation and adherence to a substrate, are down-expressed. Results of the gene expression study revealed the down-regulation of *mrkA*, involved in the initial stage of biofilm formation of *K. pneumoniae*, upon MYR treatment, while *luxS* was not involved during inhibition of mixed biofilm.

Previous literature reported that the generation of ROS could be considered as a potential cause of biofilm inhibition [42]. We found a remarkable difference in the cellular ROS profile between the MYR- treated and untreated mixed conditions confirming that both iROS and mROS accumulation was observed when the cells were challenged with MYR. The result was corroborated by the pre-treatment with two potent anti-oxidant molecules able to determine cell rescue upon MYR exposure. As reported, combination therapies reduce doses and duration of drug treatment and the associated side effects, preventing the development of resistance (Chen et al., 2021). We also tested the synergistic effect between MYR with antimicrobials through checkerboard assays. Interesting outcomes of this study are the synergistic relationship between MYR and CAS and, MYR and MEM, both displaying their effects against *C. auris*/*K. pneumoniae* biofilm at low concentrations.

Accordingly, before any possible commercial or clinical use of new drugs or their combinations, the dosage should be evaluated in vivo. Hence, from our results, it is apparent that MYR was not toxic to the invertebrate G. mellonella and also to human epithelial cells. In addition, it was able to prolong the larvae survival after infection with *C. auris*/*K. pneumoniae* and also able to protect when administered as a prophylactic agent, demonstrating a remarkable correlation between in vitro susceptibility testing results and in vivo drug efficacy in the invertebrate model. This research contributes by giving an in-depth analysis of the inhibition of biofilm formation in the mixed *C. auris/K. pneumoniae* model.

## 5. Conclusions

In conclusion, MYR showed antifungal and antibacterial activities against *C. auris* and *K. pneumoniae* planktonic cells and was also able to reduce single and mixed biofilm formation. In addition, MYR down-regulated genes related to biofilm formation and led to the induction of oxidative stress. A better performance was obtained when MYR acted in combination with two conventional drugs with a synergic effect. Future development should be addressed also to analyze biofilm eradication capacities of MYR with the combination therapy requiring more comprehensive in vivo studies before clinical application.

## Figures and Tables

**Table 1:** Gene-specific primers used for real-time RT-PCR.

Gene Name	Acronym	Primer Name	Sequence (5′→3′)
*S-ribosylhomocysteine lyase*	*luxS*	*K.pneumoniae_luxS*_F	ATCGACATTTCGCCAATGGG
		*K.pneumoniae_luxS*_R	ACTGGTAGACGTTGAGCTCC
*Type 3 fimbrial shaft*	*mrkA*	*K.pneumoniae_mrkA_*F	ACGTCTCTAACTGCCAGGC
		*K.pneumoniae_mrkA_*R	TAGCCCTGTTGTTTGCTGGT
*16S ribosomial RNA*	*16S rRNA*	*K.pneumoniae_16S_*F	AGCACAGAGAGCTTG
		*K.pneumoniae_16S_*R	ACTTTGGTCTTGCGAC
*1,3-beta-glucan synthase*	*FKS1*	*C.auris_ FKS1_*F	GCAAACTTTCATGTTGGTGTTA
		*C.auris_ FKS1_*R	TGTGAACAAGGAGTTTGAGTAA
*Ergosterol Biosynthesis*	*ERG11*	*C.auris_ERG11_*F	GTGCCCATCGTCTACAACCT
		*C.auris_ERG11_*R	TCTCCCACTCGATTTCTGCT
*Hyphal-specific genes*	*ALS5*	*C.auris_ALS5_*F	CCTTCTGGATCGGACACAGT
		*C.auris_ALS5_*R	AGTTGTGGTGGAGGAACCAG
*Actin*	*actin*	*C. auris_actin_*F	GAAGGAGATCACTGCTTTAGCC
		*C.auris_actin_*R	GAGCCACCAATCCACACAG

**Table 2:** Characterization of single- and dual-species biofilm at 24 h.

Strains	CFU/Well ± SD		% Composition Dual-Species Biofilm
	Single-Species Biofilm	Dual-Species Biofilm	
*C. auris*	(3.0 ± 0.05) × 10^6^	(6.3 ± 0.12) × 10^5^	6
*K. pneumonia*e	(6.0 ± 0.15) × 10^6^	(1.0 ± 0.03) × 10^7^	94

## Data Availability

Not applicable.

## Supplementary Materials

The following supporting information can be downloaded at: https://www.mdpi.com/article/10.3390/microorganisms10091773/s1, Table S1. MIC of myrtenol (MYR), Caspofungin (CAS) and Meropenem (MEM) against *C. auris* and *K. pneumoniae*.
