# Supplementary material for: Effect of Myrtenol and Its Synergistic Interactions with Antimicrobial Drugs in the Inhibition of Single and Mixed Biofilms of Candida auris and Klebsiella pneumoniae
Source: Microorganisms. 2022 Sep 2;10(9):1773. doi: 10.3390/microorganisms10091773 (PMC9501169; doi:10.3390/microorganisms10091773)
Supplement: Supplementary file 1 [file microorganisms-10-01773-s001.zip › microorganisms-1857576-supplementary.pdf]

microorganisms

Supplementary materials

Table S1. MIC of myrtenol (MYR), Caspofungin (CAS) and Meropenem (MEM) against *C. auris* and *K. pneumonia*e.

Strains	MYR	CAS	MEM
		MIC (μgmL^{-1}) ± SD	
C. auris DSM 24092	50.0 ± 5.0	1.0 ± 0.5	-
*K. pneumonia*e ATCC 13883	200.0 ± 20.0	-	1.0 ± 0.2
